# Chronic Maxillary Sinusitis Due to Material Compatible with Hyaluronic Filler—A Case Report

**DOI:** 10.3390/clinpract15120230

**Published:** 2025-12-08

**Authors:** Marino Lupi-Ferandin, Dinko Martinovic, Ema Puizina, Mislav Usljebrka, Andrija Rados, Lovre Martinovic, Neven Ercegovic, Josko Bozic, Slaven Lupi-Ferandin

**Affiliations:** 1 Primary Healthcare Centre of Split-Dalmatia County, 21000 Split, Croatia; 2 Department of Maxillofacial Surgery, University Hospital of Split, 21000 Split, Croatia; 3 Medical Studies, University of Split School of Medicine, 21000 Split, Croatia; 4 Department of Pathophysiology, University of Split School of Medicine, 21000 Split, Croatia

**Keywords:** hyaluronic filler, maxillary sinus, chronic sinusitis, complication, case report

## Abstract

**Background:** Chronic maxillary sinusitis is most often linked to dental, allergic, or anatomical etiologies, with foreign body-induced forms remaining rare. This case report describes a unique occurrence of chronic maxillary sinusitis resulting from misplaced hyaluronic filler due to a facial esthetic procedure. **Case presentation:** A 60-year-old woman experienced right-sided maxillary sinusitis symptoms for three years after hyaluronic filler injections. Multi-slice computed tomography showed total sinus opacification, a vermicular foreign body, and a small anterior wall perforation. The patient underwent Caldwell-Luc surgery for foreign body removal and mucosal excision, followed by histopathological analysis. **Results:** The procedure was successful, with complete extraction of the foreign body compatible with hyaluronic filler. Postoperative recovery was uneventful, and symptoms resolved. This rare complication likely resulted from accidental filler penetration into the maxillary sinus during the injection. **Conclusions:** To the best of our knowledge, after a detailed search of the available literature, this is the first reported case of chronic maxillary sinusitis caused by material that is compatible with misplaced hyaluronic filler. It stresses the critical need to minimize serious complications in the facial esthetic procedures through detailed anatomical knowledge, technical skill, and a strict credentialing protocol of practitioners. Further awareness and regulations could improve patient safety.

## 1. Introduction

Chronic maxillary sinusitis is a persistent inflammation of the maxillary sinus mucosa lasting for more than 12 weeks. It commonly manifests with symptoms such as nasal obstruction, facial pain or pressure, and purulent nasal discharge, significantly impairing patient quality of life [1]. The etiology of chronic maxillary sinusitis is multifactorial, frequently involving ongoing infections, especially odontogenic, allergic responses, or anatomical variations like septal deviation and ostiomeatal complex narrowing or obstruction [2,3]. Among these, foreign body-induced sinusitis is particularly rare but can pose substantial diagnostic and therapeutic challenges.

Foreign bodies found within the maxillary sinus are most often iatrogenic in origin, typically introduced during dental extractions, root canal treatments, or maxillofacial surgeries. Less commonly, non-dental causes such as traumatic events or accidental insertion of small objects like toothpicks or glass have been reported [4,5]. The introduction of foreign material into the sinus cavity can trigger mucosal inflammation by directly irritating the lining or obstructing the natural sinus drainage pathways, thus perpetuating or initiating chronic sinusitis. Dental materials, fractured implants, and surgical debris are among the most frequently reported sinus foreign bodies, which highlights the important interrelation of dental and sinonasal pathologies [6].

The management of chronic maxillary sinusitis often begins with conservative therapies, including antibiotics, corticosteroids, and decongestants [7]. However, the persistence of symptoms due to a retained foreign body usually requires surgical intervention. The Caldwell-Luc operation, established in the late 19th century by George Caldwell and Henri Luc, remains a time-honored approach for direct access to the maxillary sinus through the canine fossa [8]. While advances in endoscopic sinus surgery have largely supplanted open procedures for many patients, the Caldwell-Luc operation retains its relevance, particularly in complex cases where foreign bodies are extensive, inaccessible, or where direct visualization and removal are essential [9].

This report presents a unique case of chronic right maxillary sinusitis secondary to an unusual foreign body, a misplaced hyaluronic filler following a facial esthetic procedure. The documentation of such a case is exceedingly rare which stresses the need for heightened awareness of potential complications associated with esthetic treatments and underscores the importance of adequate training and regulatory oversight for these procedures.

## 2. Case Report

A 60-year-old female was referred to the Department of Maxillofacial Surgery, University Hospital of Split due to facial pain that has occurred sporadically for the past three years. After a detailed physical exam, we determined that she presented with a chronic right-sided maxillary sinusitis symptoms, including nasal congestion and postnasal drip, both persisting for three years. Her medical history revealed a facial esthetic treatment three years ago involving hyaluronic filler injections in the zygomatic and paranasal regions. She provided a medical report from the esthetic doctor with an official sticker from the package of the administrated hyaluronic filler brand and type. The patient claimed that the symptomatology regarding the cacosmia and halitosis has started a few weeks after the aforementioned esthetic procedure. Furthermore, during the last three years she was given oral antibiotics by her primary care physician on several occasions with the presumption of an acute sinusitis, but the symptomatology never fully ceased. No other significant medical history was reported.

**Figure 1:**
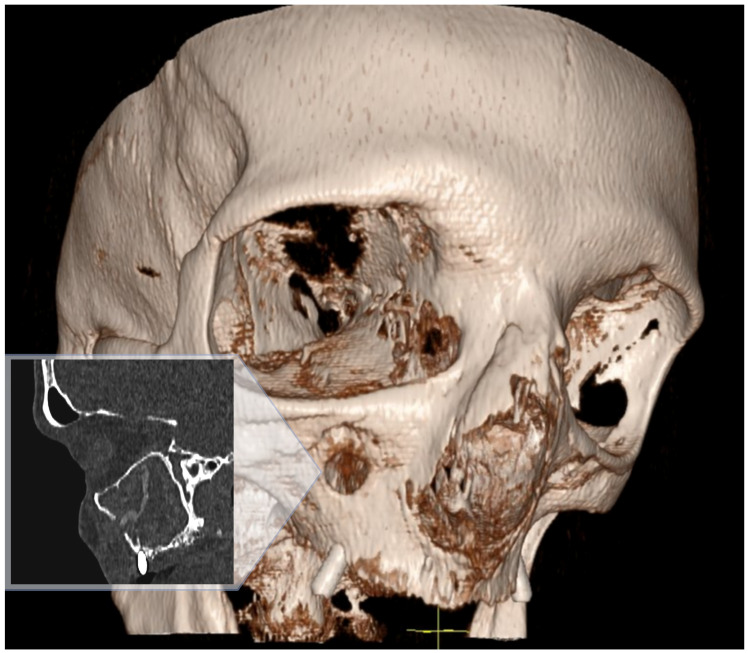
MSCT sagittal view and 3D facial bone view both show a perforation of the right maxillary sinus anterior wall, total opacification of the sinus and a foreign body inside.

Multi-slice computed tomography (MSCT) revealed mucosal hyperplasia, total opacification of the right maxillary sinus and a solid foreign body within the maxillary sinus. The foreign body had a vermicular narrow shape with an approximate length of ~28 mm. Furthermore, a small perforation of the maxillary sinus anterior wall was observed on MSCT (Figure 1).

With dental assessment it was established that the patient is edentulous with “all on four” implants and total dental prosthetics. ENT specialists were also consulted regarding the patient and they conducted nasal endoscopy with topical anesthesia; however, due to the swollen mucosa at the passage to the maxillary sinus, they did not enter the sinus. Furthermore, the ENT team was consulted regarding functional endoscopic sinus surgery (FESS). According to EPOS 2020, persistent symptoms in the presence of a retained foreign body constitute an indication for surgery [10]. In contemporary practice, endoscopic sinus surgery is the primary approach; however, in this specific case the pre-existing anterior wall perforation, the anterolateral location of the foreign body, and its partial protrusion into the soft tissues raised concern for incomplete removal via a standard endoscopic route. We therefore selected an anterior antrostomy via Caldwell-Luc, consistent with reports supporting open access when endoscopic visualization or instrumentation is insufficient for safe, complete extraction [11,12,13,14,15].

**Figure 2:**
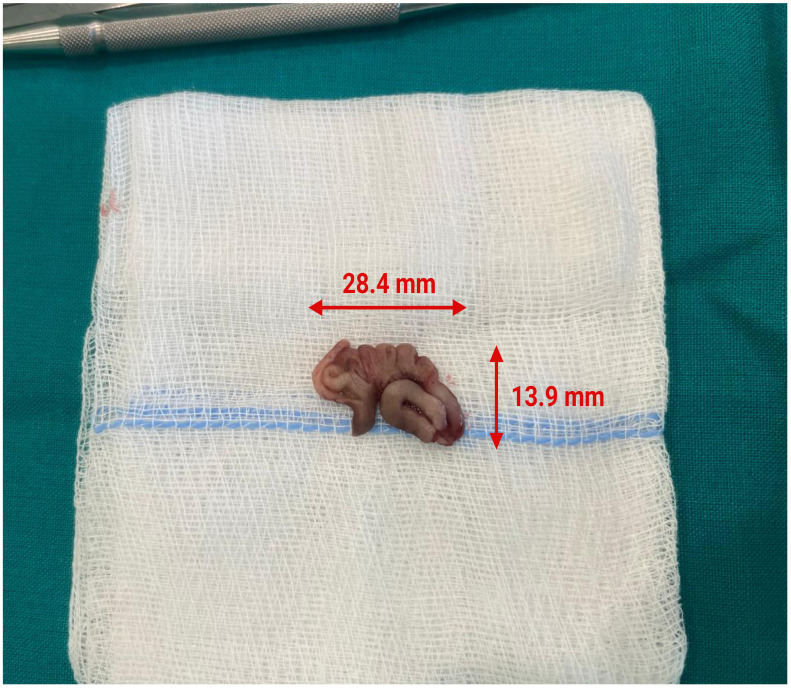
Intraoperative photo of the extracted solid vermicular foreign body with its dimensions.

Under general anesthesia, after an incision in the upper right vestibular area, a mucoperiosteal flap was elevated to expose the anterior wall of the right maxillary sinus. A small perforation (~4 mm) was identified on the anterior wall and an additional antrostomy was performed over this hole using a burr. After accessing the sinus cavity, a solid vermicular foreign body was discovered inside and removed (Figure 2). Additionally, a swab of the sinus was taken and sent to the microbiology for further analysis. Afterwards, the sinus mucosa was excised due to chronic inflammation, and the cavity was packed with iodoform tape extending to the upper right vestibule of the oral cavity. The surgical site was closed with resorbable sutures.

During the hospitalization, the patient was empirically administrated intravenous amoxicillin with clavulanic acid. The later microbiology cultures of the sinus swab reported that the accompanying bacterial infection of the sinus was caused by *Klebsiella oxytoca* and *Streptococcus mitis*, both sensitive to amoxicillin with clavulanic acid. Hence, after the report the same antibiotic therapy was continued.

The postoperative course was uneventful. The patient was discharged from the hospital 5 days after surgery and during her stay the iodoform tape, whose tip was left out in the upper oral vestibulum, was slowly pulled out. The follow-ups after discharge were once a week in the first month and during this time period the patient did not have any complications. The ENT specialist conducted a sinus endoscopy on the first and third postoperative follow-up and confirmed that the healing process is satisfactory. After the first month, the follow-ups continued once a month during the next three months and they were uneventful. All of the prior chronic sinusitis symptoms such as headaches, nasal congestion, cacosmia and halitosis have resolved.

**Figure 3:**
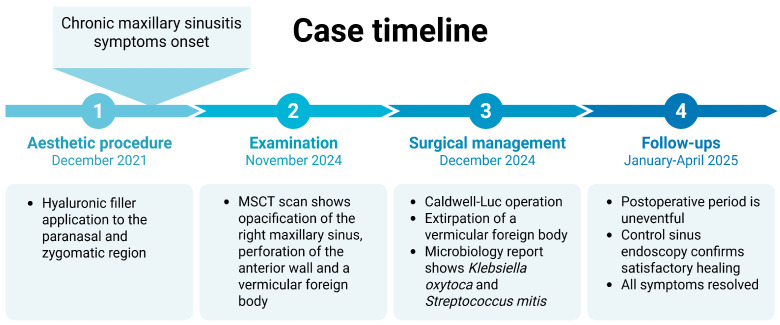
Timeline chart of the case.

The extracted foreign body was sent for further pathohistological analysis. However, since the filler was not actually inside the tissue but was essentially exposed to air in the sinus cavity, it dried out during the three years and calcified under the prolonged exposure of air. Hence, it was more like a solid “foreign body” and due to that, the pathologist was unable to cut it for more detail analysis and photographs. Considering its composition, shape, location, and patient history, the pathologist deducted that it was most likely a hyaluronic filler. Given that conventional histology was not feasible, we have adopted conservative wording and refer to the extracted material as compatible with hyaluronic acid filler, while acknowledging this limitation in the interpretation of causality. We concluded that it was probably inadvertently applicated through the anterior wall into the maxillary sinus during the patients’ prior esthetic treatment. A timeline chart shows the chronology of the case management (Figure 3).

## 3. Discussion

Foreign body-induced chronic maxillary sinusitis, though uncommon, is a great example of the complex anatomy in the facial region. Most documented foreign body cases are dental in origin, resulting from the unintentional displacement of tooth roots, dental impression material, or fractured dental instruments during endodontic procedures or tooth extractions [6]. Non-dental foreign bodies, such as those caused by facial trauma or iatrogenic medical errors, appear rarely in the literature [16,17]. Foreign bodies within the maxillary sinus can remain asymptomatic for extended periods of time or may manifest only after secondary infection or chronic inflammation develops. The chronic presence of foreign material can provoke persistent mucosal changes and promote bacterial colonization with the obstruction of the normal mucociliary clearance [18]. This consequently perpetuates a cycle of ongoing sinusitis. Imaging modalities such as MSCT are invaluable in identifying atypical contents within the sinus and mapping out surgical access routes. While endoscopy is setting the golden standard regarding the procedures on the sinuses, conversely it is deemed that the open surgical intervention remains the cornerstone for managing chronic sinusitis secondary to foreign bodies, especially when conservative or less invasive measures fail [11]. The size and anatomical location of foreign bodies represent critical factors in determining the optimal surgical approach for removal from the maxillary sinus. Small foreign bodies, particularly those with minimal dimensions, are ideally suited for endoscopic removal, as these can be visualized and extracted through the natural ostium or modest middle meatal antrostomy with minimal tissue disruption [12,13]. Conversely, large or bulky foreign bodies present significant technical challenges for conventional endoscopic approaches, necessitating consideration of extended endoscopic access or conversion to open surgical techniques [13]. Foreign bodies located on the sinus floor or anteroinferior regions are challenging to access via conventional endoscopic middle meatal antrostomy due to limited visualization and instrument maneuverability [14]. Furthermore, freely mobile foreign bodies tend to be more amenable to endoscopic retrieval, as they may migrate distally during the procedure and be more easily extracted under direct visualization. In contrast, impacted or fixed foreign bodies closely adherent to the sinus mucosa or lodged against sinus walls may necessitate wider surgical access for safe removal without mucosal damage [14]. Even though it is more invasive and has a higher degree of possible complications, Caldwell-Luc operation is still highly effective and its approach allows for direct access and complete removal of foreign material as well as meticulous debridement of chronically inflamed tissue, particularly in cases where endoscopic visualization is limited due to the size, shape, or location of the foreign body [15]. In the present case, the Caldwell-Luc operation facilitated successful resolution of the patient’s chronic symptoms and prevented further complications, while also there were no complications from the approach itself. From a surgical standpoint, endoscopic sinus surgery is the standard first-line technique in the endoscopic era, with open approaches such as Caldwell-Luc reserved for rare situations where endoscopic access is unlikely to achieve safe and complete removal; our case met these criteria due to the foreign body’s anterolateral position and partial extra-sinus protrusion.

Facial esthetic interventions, particularly the use of injectable fillers such as hyaluronic acid, have become increasingly popular due to their minimally invasive nature, versatility, and perceived safety profile [19,20]. Nevertheless, these procedures are not devoid of risk. Complications range from transient local reactions such as erythema, edema, pain, bruising, and tenderness to more severe outcomes including infection, delayed-onset nodules, foreign body granuloma formation, vascular compromise, and tissue necrosis [21]. While most adverse events are mild and self-limiting, on the other hand life-altering complications such as arterial embolic obstruction and subsequent skin necrosis or even vision loss have also been reported and are often linked to poor technique, anatomic misjudgment, or lack of injector expertise [22,23,24]. There is strong evidence of correlation between complication rates and practitioner experience: procedures performed by inexperienced or non-medical personnel exhibit a significantly greater incidence of both ischemic and non-ischemic complications compared to those conducted by certified clinicians [23].

Critical factors influencing these risks include the injector’s familiarity with facial vascular anatomy, choice of injection site, depth and placement, and whether precautionary protocols—such as aspiration and slow injection—are rigorously followed [25]. The nasolabial fold and nose, both highly vascularized regions, are particularly prone to ischemic complications when injections are administered haphazardly or by those lacking anatomical knowledge. Conversely, technical errors such as superficial placement or excessive filler volume can lead to contour irregularities, delayed-onset nodules, and granulomatous inflammatory responses. Patient-related factors, including smoking status and underlying dermatological disease, may modulate risk, but operator experience remains the decisive determinant in complication rate. Despite the overall favorable safety record of facial fillers, it is notable that no amount of experience can guarantee absolute immunity from adverse events, as unique anatomical variations and product characteristics may contribute unpredictably to outcomes [26]. Nonetheless, diligent adherence to evidence-based technique and ongoing education dramatically mitigates the likelihood and severity of complications. Regular refresher training and strict credentialing for practitioners should therefore be prioritized, with regulatory oversight to ensure high standards of patient safety. Ultimately, minimization of filler-related complications in the facial area is most reliably achieved by those with deep anatomical knowledge, sound clinical judgment, and commitment to procedural mastery.

This case is unique in demonstrating direct penetration of filler material into the maxillary sinus, likely through an accidental breach of the anterior wall during the injection. While the complication was unintentional it also underscores the importance of anatomical knowledge and technical expertise during this kind of esthetic procedures. We believe that esthetic procedures require profound understanding of the complex facial anatomy which is essential for safe filler administration by reducing the risk of inadvertent intrusions into vital structures. Additionally, with the growing demand for minimally invasive esthetic interventions, it is imperative that only appropriately trained healthcare professionals perform these procedures. Regulatory bodies should ensure strict credentialing and oversight to maintain high standards of patient safety. Hence, even though complications can also happen to every professional, the prompt identification and management of the procedural complications, including unusual presentations like foreign body-induced sinusitis, can significantly affect clinical outcomes [27].

After comprehensive literature search in PubMed, Scopus, and Web of Science up to 6 September 2025 no prior case report describing chronic maxillary sinusitis due to misplaced hyaluronic filler in the maxillary sinus was identified. The search was conducted using the terms and combinations: “hyaluronic acid” OR “hyaluronic filler” AND “maxillary sinus” AND (sinusitis OR foreign body OR complication), including relevant MeSH terms for hyaluronic acid, dermal fillers, maxillary sinus, paranasal sinus diseases, and foreign bodies.

The limitation of this case report is the occurrence that histopathology was unable to analyze the extracted material. As previously explained, since the “filler” was not injected inside the tissue but actually exposed to air inside the sinus cavity, it dried out in this conditions and calcified. Hence, the diagnosis of the hyaluronic filler as the foreign body is anamnestic and clinical.

## 4. Conclusions

To the best of our knowledge, and after a detailed search of the available literature, there were no previous reported cases of chronic maxillary sinusitis caused by material that is compatible with misplaced hyaluronic filler. Further research and documentation of similar cases could improve understanding and prevention of such complications. Furthermore, this report not only presents a rare and instructive complication following an esthetic procedure but also reinforces the critical importance of comprehensive medical training and robust procedural standards in the rapidly expanding field of facial esthetics.

## Data Availability

The original contributions presented in this study are included in the article. Further inquiries can be directed to the corresponding author.
